# Phenolic Acids Rescue Iron-Induced Damage in Murine Pancreatic Cells and Tissues

**DOI:** 10.3390/molecules28104084

**Published:** 2023-05-14

**Authors:** Tugba Kose, Paul A. Sharp, Gladys O. Latunde-Dada

**Affiliations:** Department of Nutritional Sciences, School of Life Course and Population Sciences, King’s College London, Franklin-Wilkins-Building, 150 Stamford Street, London SE1 9NH, UK; tugba.kose@kcl.ac.uk (T.K.); paul.a.sharp@kcl.ac.uk (P.A.S.)

**Keywords:** iron overload, pancreatic beta cells, ROS, antioxidant activity

## Abstract

Iron is an essential element involved in a variety of physiological functions. However, excess iron catalyzes the generation of reactive oxygen species (ROS) via the Fenton reaction. Oxidative stress, caused by an increase in intracellular ROS production, can be a contributory factor to metabolic syndromes such as dyslipidemia, hypertension, and type 2 diabetes (T2D). Accordingly, interest has grown recently in the role and use of natural antioxidants to prevent iron-induced oxidative damage. This study investigated the protective effect of the phenolic acids; ferulic acid (FA) and its metabolite ferulic acid 4-O-sulfate disodium salt (FAS) against excess iron-related oxidative stress in murine MIN6 cells and the pancreas of BALB/c mice. Rapid iron overload was induced with 50 μmol/L ferric ammonium citrate (FAC) and 20 μmol/L 8-hydroxyquinoline (8HQ) in MIN6 cells, while iron dextran (ID) was used to facilitate iron overload in mice. Cell viability was determined by 3-(4,5-dimethyl-2-thiazolyl)-2,5-diphenyltetrazolium bromide (MTT) assay, ROS levels were determined by dihydrodichlorofluorescein (H2DCF) cell-permeant probe, iron levels were measured by inductively coupled plasma mass spectrometry (ICP-MS), glutathione, SOD (superoxide dismutase) and lipid peroxidation, and mRNA were assayed with commercially available kits. The phenolic acids enhanced cell viability in iron-overloaded MIN6 cells in a dose-dependent manner. Furthermore, MIN6 cells exposed to iron showed elevated levels of ROS, glutathione (GSH) depletion and lipid peroxidation (*p* < 0.05) compared to cells that were protected by treatment with FA or FAS. The treatment of BALB/c mice with FA or FAS following exposure to ID increased the nuclear translocation of nuclear factor erythroid-2-related factor 2 (Nrf2) gene levels in the pancreas. Consequently, levels of its downstream antioxidant genes, HO-1, NQO1, GCLC and GPX4, increased in the pancreas. In conclusion, this study shows that FA and FAS protect pancreatic cells and liver tissue from iron-induced damage via the Nrf2 antioxidant activation mechanism.

## 1. Introduction

Iron is a potent pro-oxidant and has a fined-tuned control mechanism that regulates iron entry, iron recycling and iron distribution within the body iron [1]. There are no specific excretory pathways for iron so if excess iron is stored in the body there is a high risk of iron overload and iron-related toxicity. Evidence abounds that increased levels of iron can induce the generation of reactive oxygen species (ROS) via the Fenton reaction. This forms the basis for iron-induced pancreatic β-cell apoptosis and dysfunction in humans [2,3] and in mouse models of hereditary hemochromatosis [4]. The Fenton reaction involves iron-induced catalytic degradation of hydrogen peroxide into a highly reactive hydroxyl free radical which attacks pancreatic β cells through increased oxidative stress and induces β cell apoptosis [5].

Pancreatic islets are highly vulnerable to iron overload-mediated oxidative stress and can lead to impaired insulin signalling, mitochondrial dysfunction, and inhibition of ATP production [6,7]. Iron overload affects several other tissues involved in lipid and glucose metabolism such as the muscle, liver, adipose tissue, and organs influenced by chronic diabetic complications [8]. For example, excess iron may diminish glucose utilization in muscle and lead to a shift from glucose to fatty acid oxidation and may result in increased insulin resistance [9]. In addition, increased iron storage in the liver may induce insulin resistance by decreasing hepatic insulin extraction from the circulation [10]. Moreover, elevated total body iron is associated with an increase in the rate of adipocyte lipolysis and higher circulating free fatty acid levels, which in turn mediates insulin resistance and raises the risk of type 2 diabetes [11].

Pancreatic islets are extremely susceptible to oxidative stress, possibly due to low expression of the antioxidant defence enzymes and the exclusive reliance on the mitochondrial metabolism of glucose [12]. Iron-induced oxidative stress can result in pancreatic cell damage. Death of β cells can consequently lead to diminished insulin secretion and therefore the development of T2D [13]. Mouse models of HFE-associated hereditary hemochromatosis (HFE-HH), which are characterised by excessive intestinal absorption and accumulation of iron in several tissues (including the pancreas), show increased expression of apoptotic genes in the pancreas [4]. A meta-analysis of 11 prospective studies including patients with hereditary hemochromatosis (HH) showed that increased body iron stores were considerably associated with a greater risk of T2D [9]. Consequently, it is thought that iron accumulation is likely related to the development of insulin resistance and T2D.

In the past decades, plant-origin phenolic acids have drawn increasing scientific attention due to their potential antioxidant properties and their vital effects in preventing various oxidative stress-associated diseases [14]. Two mechanisms are commonly proposed to explain the multifunctional antioxidant role of phenolic compounds, namely: metal chelation and free radical scavenging [15]. Phenolic acids are thought to play a naturally protective role in excess iron-related diseases and iron-induced ROS generation. Ferulic acid (FA) is the most abundant phenolic acid found in cereal grains, which constitutes its primary dietary source. FA is more easily absorbed into the body and stays in the blood longer than any other phenolic acid; thus, it is a superior antioxidant [16]. FA has low toxicity and possesses several physiological functions, including anti-inflammatory, antimicrobial, anticancer anti-arrhythmic and antithrombotic activity. It also has demonstrated antidiabetic effects and immunostimulant properties, and it reduces nerve cell damage and may repair damaged cells [16].

In the present study, we investigated iron-induced oxidative damage in murine MIN6 cells and the pancreas and examined the accompanying changes in antioxidant status. In addition, we tested whether FA treatment might protect pancreatic β-cells and tissues against excess iron by activating the Nrf2 antioxidant mechanism.

## 2. Results

### 2.1. Protective Effects of Phenolic Acids against Iron Overload in MIN6 Cells

**Figure 1:**
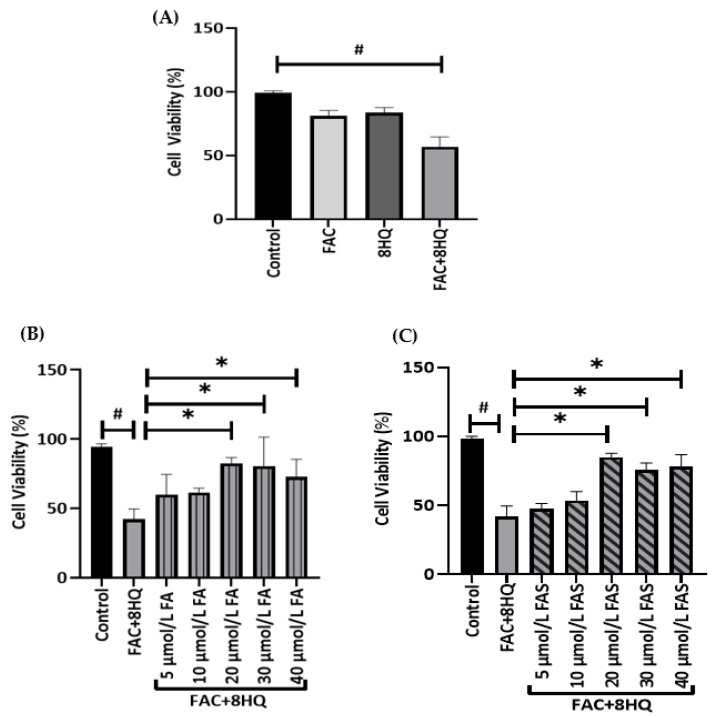
Protective effects of phenolic acids against iron-mediated toxicity in MIN6 cells. Cells were exposed to FAC and/or 8HQ for 2 h to assess the effect of iron overload (**A**). MIN6 cells were treated with different concentrations of FA (**B**) or FAS (**C**) overnight and then exposed to FAC + 8HQ for 2 h. Data are expressed as percentage cell viability relative to control cell samples; # *p* < 0.05 control vs. FAC + 8HQ group only, * *p* < 0.05 FAC + 8HQ group only vs. treatment groups. One-way ANOVA, Tukey post hoc test.

**Figure 2:**
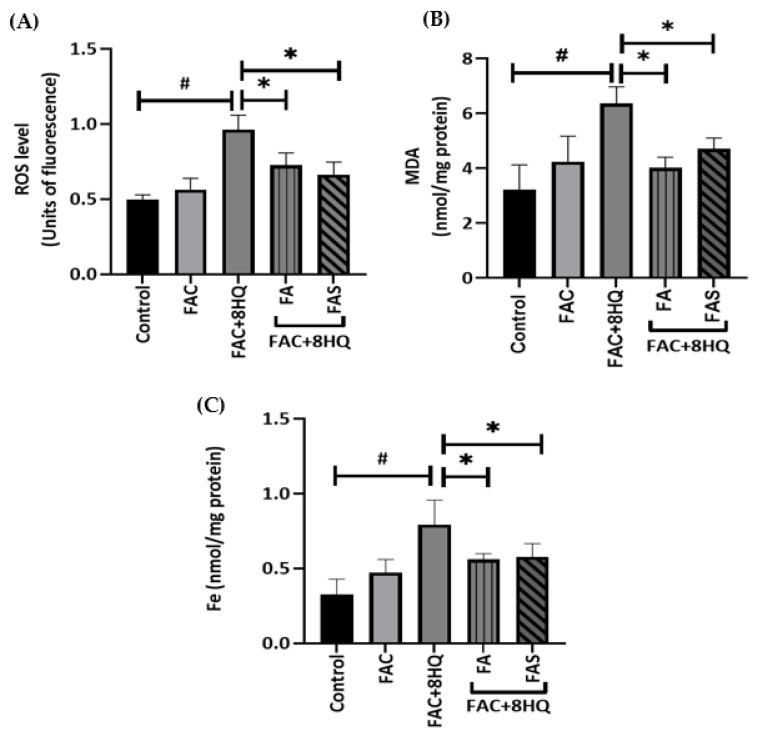
FA and FAS limit lipid peroxidation, ROS production and iron accumulation in iron overloaded MIN6 cells. MIN6 cells were treated with or without 20 μmol/L FA or FAS overnight and then exposed to FAC + 8HQ for 2 h. Cellular reactive oxygen species (ROS) was estimated with dihydrodichlorofluorescein (H2DCF) cell-permeant probe (**A**), lipid peroxidation levels were assessed by Malondialdehyde (MDA) assay (**B**) and iron concentrations were measured by inductively coupled plasma mass spectrometry (ICP-MS) (**C**); # *p* < 0.05 control vs. treatment groups, * *p* < 0.05 FAC + 8HQ group only vs. treatment groups. One-way ANOVA, Tukey post hoc test.

To examine whether phenolic acids could protect pancreatic cells under iron overload conditions, rapid iron loading in cells was achieved by treating MIN6 cells with 50 μmol/L FAC and 20 μmol/L 8HQ (8HQ + FAC) for 2 h [17]. The FAC + 8HQ complex is an established model to study iron-induced toxicity in cells; 8HQ is a lipophilic chelator that facilitates the rapid entry of iron into cells, and it exerts toxicity to cells even after 15 min [17,18]. This has been demonstrated in other cellular models [17,19]. Iron bound to the lipophilic iron chelator, 8-hydroxyquinoline, causes DNA strand breakage in cultured lung cells [20]. Consequently, cell viability was reduced by 54% (*p* < 0.05) following FAC + 8HQ treatment compared with the control group (Figure 1A). Next, to examine the protective effect of FA and FAS treatments on iron overload in MIN6 cells, cells were treated with or without FA and FAS (5, 10, 20, 30 and 40 μmol/L) overnight before exposure to FAC + 8HQ. Concentrations of phenolic acids were determined based on the antioxidant effects of FA [21]. The decrease in cell viability following exposure to FAC + 8HQ was abolished by FA or FAS treatment in a dose-dependent manner (Figure 2B, C; *p* < 0.05). The maximal effective concentration of both phenolic acids was 20 μmol/L and, therefore, this concentration of FA and FAS was selected for further experiments.

### 2.2. Depletion of Oxidative Stress Markers in MIN6 Cells Treated with Phenolic Acids

To demonstrate whether FA and FAS might protect cells from iron overload, pancreatic MIN6 cells were treated overnight with or without 20 μmol/L FA or FAS and then exposed to FAC + 8HQ for 2 h. Intracellular ROS level was detected in FAC + 8HQ-treated MIN6 cells by measuring 2′, 7′–dichlorofluorescein (DCF) by flow cytometry. Compared with the control group, the intracellular ROS level of MIN6 cells significantly increased following treatment with FAC + 8HQ (*p* < 0.05). Notably, pre-treatment with FA and FAS effectively reduced FAC + 8HQ-induced ROS accumulation (Figure 2A), although treatment with FA or FAS in the absence of FAC + 8HQ did not change intracellular ROS levels in MIN6 cells [22]. MDA, a product of lipid peroxidation, indirectly reflects the degree of disruption in the cell membrane [23]. In order to indicate the protective capacity of phenolic acids against lipid peroxidation, MDA levels were measured in MIN6 cells. When compared with the FAC+8HQ treatment group, MDA levels in iron-loaded cells following FA or FAS treatment were significantly decreased (*p* < 0.05; Figure 2B). MIN6 cells did not show any significant change in MDA levels when treated with 50 μmol/L FAC only, attesting to the efficacy of 8HQ to effect rapid diffusion of iron into the cells (Figure 2). To clarify whether FA and FAS have inhibitory effects against iron accumulation, cellular iron levels were measured by inductively coupled plasma mass spectrometry (ICP-MS). FAC alone resulted in a modest but insignificant increase in cellular iron. In comparison, FAC + 8HQ treatment group showed a significant increase in iron levels in comparison to the control group (*p* < 0.05; Figure 2C). Other forms of iron also increased cellular iron levels; however, FAC + 8HQ was the most effective (Figure S1). The increase in cellular iron following FAC + 8HQ was reversed by pre-treatment of MIN6 cells with FA or FAS by 0.6 and 0.7 nmol/mg protein, respectively (*p* < 0.05; Figure 2C).

### 2.3. Cellular Antioxidant Activity in MIN6 Cells Exposed to Phenolic Acids

**Figure 3:**
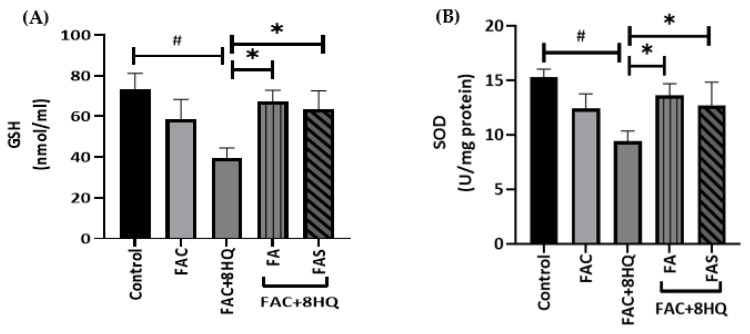
Effects of FA and FAS on the antioxidant activity in MIN6 cells. Pancreatic MIN6 β cells were treated with 20 μmol/L of FA or FAS overnight and then exposed to FAC + 8HQ for 2 h. Glutathione (GSH) levels (**A**) and superoxide dismutase (SOD) activity (**B**) levels were measured; # *p* < 0.05 control vs. treatment groups, * *p* < 0.05 8HQ + FAC group only vs. treatment groups. One-way ANOVA, Tukey post hoc test.

Recent studies have shown that the imbalance in the redox state is involved in pancreatic β cell damage [24]. FAC + 8HQ complex can penetrate rapidly through cellular membranes due to the increased lipophilic nature of this metal complex. In addition, 8HQ results in a high-stability iron complex, which leads to rapid iron accumulation and ROS production [25]. As both phenolic acids effectively reduced ROS levels in FAC + 8HQ-treated MIN6 cells, it was speculated that the effect of FA or FAS treatments could enhance the antioxidant activity in pancreatic β cells supplemented with iron. To test this notion, GSH levels and SOD activity were measured in MIN6 cells treated overnight with (and without) 20 μmol/L FA or FAS and subsequent exposure to FAC + 8HQ for 2 h. As expected, FAC + 8HQ-treated MIN6 cells displayed impaired GSH and SOD activity by 59% and 57% (*p* < 0.05), respectively, while the pretreatment with FA or FAS significantly diminished the iron-induced decrease in GSH and SOD (Figure 3A,B; *p* < 0.05). Phenolic acids alone did not show any significant effects on the activities of the enzymes in MIN6 pancreatic cells, as shown in our previous study [22].

### 2.4. Insulin Secretion Is Increased in MIN6 Cells Treated with Phenolic Acids

**Figure 4:**
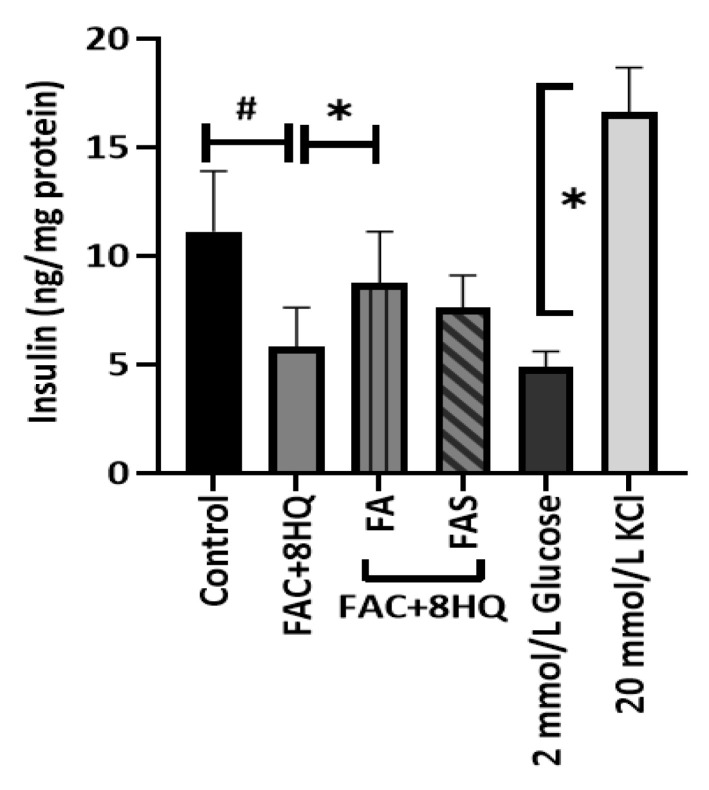
Phenolic acids and iron overload conditions affect insulin secretion in MIN6 β cells. Mouse MIN6 β cells were treated with 20 μmol/L ferulic acid (FA), ferulic acid 4-O-sulfate disodium salt (FAS) and with/without FAC + 8HQ for 3 h under 20 mmol/L glucose conditions. Supernatants from triplicate MIN6 cell samples were analyzed for insulin secretion; # *p* < 0.05 controls vs. treatment groups, * *p* < 0.05 8HQ + FAC group only vs. treatment groups and * *p* < 0.05 2 mmol/L glucose treatment vs. 20 mmol/L KCl treatment groups. One-way ANOVA, Tukey post hoc test.

In line with its cytotoxic effects, rapid iron overload treatment significantly decreased insulin secretion (*p* < 0.05) in MIN6 cells. This effect was reversed by FA treatment and in the case of FA there was a significant 0.5-fold increase in insulin secretion compared with the FAC + 8HQ treatment group (Figure 4) (*p* < 0.05). Under similar experimental conditions, the depolarizing agent KCl (20 mmol/L) as a positive control induced a 4.5-fold increase in insulin secretion compared with the low glucose level group (2 mmol/L) (*p* < 0.05).

### 2.5. Phenolic Acids Activate Nrf2 Signaling Pathway in Pancreatic Tissues under Iron Overloaded Conditions

**Figure 5:**
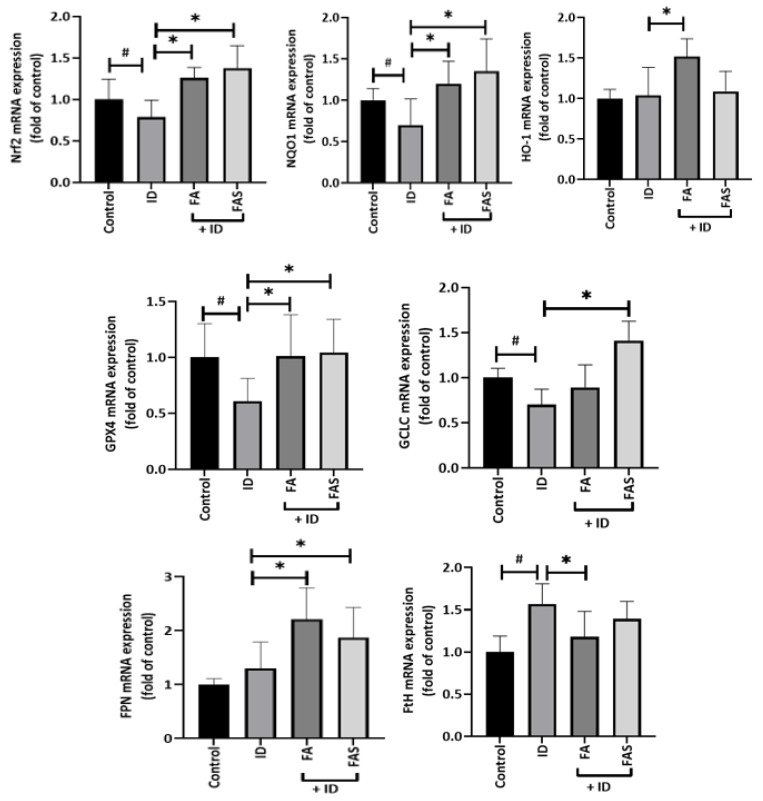
Expression of Nrf2 antioxidant pathway-related genes in the pancreas. The mRNA levels of nuclear factor erythroid 2-related factor 2, Nrf2, NAD(P)H Quinone Dehydrogenase 1 (NQO1), glutamate-cysteine ligase catalytic subunit (GCLC), haem oxygenase-1 (HO-1), glutathione peroxidase 4 (GPX4), iron storage protein ferritin H (FtH) and ferroportin (FPN) were quantified by RT-PCR analyses in pancreatic tissues of BALB/c male mice exposed to 0.1% DMSO (Control), iron dextran (100 mg/kg) (ID), ferulic acid (20 mg/kg)(FA) or ferulic acid 4-O-sulfate disodium salt (20 mg/kg) (FAS) with ID (n = 5 mice per group). Gene expression was determined by qPCR 2^−ΔΔCT^ in the pancreas and normalized with housekeeping gene, RPL19; # *p* < 0.05 DMSO vs. treatment groups, * *p* < 0.05 ID group only vs. treatments groups. One-way ANOVA, Tukey post hoc test.

To further identify whether the protective effects of FA and FAS on the pancreas under iron-overloaded conditions are dependent on the Nrf2 pathway, the mRNA levels of Nrf2 and some of its downstream genes were determined in pancreatic tissues of iron-loaded BALB/c mice with or without administration of phenolic acids. To facilitate iron accumulation, iron dextran (100 mg/kg) (ID) was injected intraperitoneally in mice on alternate days for 10 days. Mice were given FA and/or FAS for 10 consecutive days (20 mg/per kg of FA or FAS given by oral gavage (100 μL volume)). Nrf2, NQO1, GCLC, HO-1 and GPX4 mRNA expression were determined by qPCR. As is shown in Figure 5, Nrf2, NQO1, GCLC, HO-1 and GPX4 gene expressions in pancreatic tissues of mice treated with phenolic acids were significantly elevated (four-fold) compared with mice treated with iron dextran (ID) only (*p* < 0.05). Additionally, to understand the consequence of iron overload and deposition in pancreatic tissues, mRNA expression of FPN levels, the only known efflux protein, was determined (Figure 5). ID alone had no effect on FPN mRNA levels. However, treatment with FA or FAS under ID conditions increased the mRNA expressions of FPN levels by 1.5- and 1.2-fold, respectively. To determine whether these changes in mRNA expression following ID and/or phenolic acid treatment might relate to cellular iron levels we measured mRNA expression of the iron storage protein ferritin (FtH) which is strongly regulated by cellular iron status. FtH mRNA was significantly increased in the pancreas of iron-treated mice (*p* < 0.05), while FA supplementation in ID-treated mice reversed this effect significantly (*p* < 0.05).

## 3. Discussion

Experimental and clinical studies suggest the involvement of oxidative stress in the development and progression of metabolic syndrome and its complications in disorders such as T2D, hyperglycemia and coronary heart disease [26,27]. The therapeutic antioxidant potentials of phenolic acids in preventing oxidative stress-related diseases have been considered in numerous studies [28,29]. FA has thus been well-characterised as a phenolic compound with antioxidative and anti-inflammatory properties that can exert a protective role against oxidative stress [30]. This study investigated whether the phenolic acid FA, or its metabolite FAS, play the role of inhibitors of iron-induced oxidative stress in pancreatic β cells and tissues.

The current study used MIN6 cells as an in vitro model that possesses both glucose-induced insulin secretion capacity and low antioxidant activity. Pancreatic β cells are sensitive to oxidative stress due to the high production of ROS during metabolism and relatively low levels of antioxidant enzymes, particularly those associated with GSH metabolism [31]. A model of iron-induced damage was employed in which MIN6 cells were exposed to 50 μmol/L FAC and 20 μmol/L 8HQ for 2 h. The FAC + 8HQ protocol is a rapid iron-loading model with which to study iron-dependent ROS induction, oxidative stress, and insulin resistance [17]. This approach has been used previously to elucidate the effect of curcumin against iron-induced cell damage [17].

Oxidative stress represents an imbalance between ROS production and antioxidant defense capacity [32]. Damage to antioxidant mechanisms can cause excessive accumulation of ROS, leading to oxidative injury [33]. Iron-induced oxidative stress is closely correlated with several chronic diseases. Here, we showed that in MIN6 cells, FA could reduce cellular iron, improve cell viability, decrease ROS production and MDA levels and increase GSH and SOD activity following an iron-induced oxidative challenge. Consistent with these findings, FA treatment has been shown to reduce MDA levels and increase GSH levels in sepsis-induced rats, suggesting FA can counteract the effects of sepsis-induced oxidative damage [31]. The mechanism of action of FA is based on the inhibition of the formation of reactive oxygen species (ROS), and chelation of metal ions [30]. FA has an OH group on its aromatic ring, which has the capacity to bind metal ions including iron. This chelating effect might reduce the amount of free iron and thus the capacity to generate free radicals [34].

Our results showed that excess iron levels in MIN6 pancreatic cells reduced glucose-stimulated insulin secretion levels compared to the control group. This was partly rescued by treatment with FA (Figure 4). A study by Nomura et al. observed a similar trend where 10 μmol/L FA significantly increased glucose-stimulated insulin secretion in rat pancreatic RIN-5F cells [35]. Iron overload has been associated with impaired insulin secretion, insulin signaling, and glucose metabolism [36]. The association may be bidirectional as hyperinsulinemic conditions induce the redistribution of transferrin receptors on the cell surface, thereby enhancing tissue iron storage [37]. Although there is conflicting evidence for an association between hyperferritinemia and T2D, mild elevations in body iron stores have been shown to correlate with the onset of aberrations in glucose metabolism [38]. Despite regulated mechanisms for maintaining iron homeostasis [27], excess iron levels can deplete cellular energy stores through oxidative stress, thus leading to cell death and insulin insufficiency [39]. This may imply that severe iron overload could contribute to the aetiology of T2D. A study by Abraham et al. showed that HH subjects had a low insulin secretion capacity, whereas normalization of iron deposition with the phlebotomy technique improved insulin secretory capacity [40]. Further studies are required to determine whether phenolic acids can play an antidiabetic role in conditions where iron overload is prevalent.

Previous studies have shown that iron accumulation in the liver of Swiss albino mice following iron dextran treatment led to increased production of MDA and ROS. Supplementation with phenolic acid, tannic acid, decreased liver iron, MDA and ROS levels [41]. Natural compounds including phenolic acids exhibit not only a direct antioxidant mechanism by removing ROS but may also impact an endogenous protective mechanism such as the Nrf2 defense signaling pathway [42]. Nrf2 is the central regulator involved in maintaining cellular redox homeostasis and modulating the expression of phase II enzymes such as NQO1, GCLC, GPX4 and HO-1, which in turn protect cells from oxidative damage, inflammation, and apoptosis [43]. Previous studies have confirmed that Nrf2 activation restores the impaired antioxidant signaling to resist oxidative stress and the enhancement of Nrf2 activity is, therefore, a potential therapeutic strategy for the treatment of oxidative stress-induced diseases [44,45]. In the present study, FA and FAS treatment effectively upregulated Nrf2 and its downstream antioxidative genes at the transcriptional level in pancreatic tissues of iron-loaded BALB/c male mice. A limitation of our study was the lack of available pancreatic tissue to perform a confirmatory analysis of protein expression levels. Nonetheless, our data provide evidence that the antioxidant effect of FA on iron-loaded cells and pancreatic tissues is achieved by activating the Nrf2 pathway. Interestingly, FA treatment increased mRNA expression of the iron exporter FPN, which may limit iron accumulation in the pancreas. To determine whether the FA-mediated changes in Nrf2-regulated genes were associated with pancreatic iron status, we measured the mRNA levels of the iron storage protein FtH. The expression of FtH is highly sensitive to changes in tissue iron content and, in our studies, FtH was increased by ID supporting the notion that ID treatment increases pancreatic iron levels [46]. When mice were co-treated with FA, there was a significant decrease in FtH expression. Given the iron chelating effects of FA noted in MIN6 cells (Figure 2C), the suppression of iron-induced FtH mRNA by FA most likely occurs through reducing iron storage in the pancreas.

The doses of FA and FAS (20 mg/kg) used in our animal study were calculated according to Reagan-Shaw et al. [47]. The human equivalent dose for the mice is 1.6 mg/kg, which corresponds to around 112 mg of FA consumed daily by adults [48]. Although there have been few studies on the tissue distribution of FA after oral administration, FA remains in circulation longer than other antioxidants such as ascorbic acid, which are cleared rapidly [49]. FA may therefore be available for use by a wider range of tissues to combat changes in oxidative metabolism.

In summary, FA can protect pancreatic cells and tissues against iron-induced oxidative stress damage through Nrf2 signaling defense mechanism. The phenolic acids perform this function by metal chelation, free radical scavenging and potentially by activation of the Nrf2 antioxidant signaling pathway. The implications of these findings to the management of iron overload damage oxidative stress disorders require further investigation.

## 4. Materials and Methods

Experimental reagents and antibodies: Phenolic acids, ferulic acid (FA) and ferulic acid 4-O-sulfate disodium salt (FAS) were purchased from Toronto Research Chemicals (Toronto, ON, Canada). All other reagents were procured from Sigma-Aldrich (Dorset, UK), unless specified. Most of the methods employed in the current study are standard operating procedures, as described in our previous studies [22,50].

MIN6 cells: The mouse MIN6 pancreatic β-cell line was used in this study for maintaining Dulbecco’s modified Eagle’s medium (DMEM) [51]. The cells (<30 passages) were routinely cultured in T25-cm^2^ plastic flasks. DMEM was supplemented with 0.1 mg/mL streptomycin, 15% heat-inactivated fetal bovine serum (FBS) and 100 U/mL penicillin. Cells were kept at 37 °C under a humidified incubator in the presence of 5% CO_2_. The medium was changed twice a week. Cells were used for experimentation or split when 80–90% confluent.

Experimental groups: Six-week-old BALB/c male mice (24–30 g each) were housed at 21–23 °C in a 12-h dark/light cycle. The mice were nourished with a standard laboratory pellet diet and water ad libitum. Animal care and all procedures were conducted in accordance with methods approved by the College Research Ethics Committee of King’s College London, and the United Kingdom Animals (Scientific Procedures) Act. 1986 (ASPA). Animals were divided randomly into four groups *(n* = 5): control, iron dextran (ID), FA + ID and FAS + ID. Iron treatment received five doses of ID injected intraperitoneally (i.p.) every 2 days for 10 days to induce the iron overload condition. Mice were exposed to FA and/or FAS for 10 days by gavage a day before the commencement of iron loading. The control group mice were given sterile saline by gavage for 10 days. Euthanasia of the mice was carried out on the 11th day by injecting 0.4–0.6 mL of pentobarbitone sodium 20% *w/v* solution (i.p.) into the mice. Pancreatic tissues were collected and flash-frozen with liquid nitrogen and kept at −70 °C.

Cell Viability Assay: Protective effects of phenolic acids against iron-induced cell death were investigated in pancreatic MIN6 cells according to the protocol described in our previous study (22). Cellular metabolic activity was measured using the 3-(4,5 dimethyl-2-thiazolyl)-2,5-diphenyltetrazolium bromide (MTT) assay in a 96-well plate. MIN6 cells were cultured at a density of 5 × 10^4^ cells per well and pre-treated with different concentrations of FA or FAS overnight, before being treated with 8HQ + FAC for 2 h. After different treatments, cells were washed with fresh PBS to remove supernatants. An amount of 10 μL of MTT solution (5 mg/mL in sterile phosphate buffer saline) and 100 µL of fresh DMEM were added to each well. Then, incubating for 3 h at 37 °C, 100 μL of a solubilization buffer, dimethyl sulfoxide (DMSO) was added and incubated for 15 min at room temperature. To determine the MTT reaction in the cells, optical density was read in a microplate reader (Bio-Tek ELx800, Bio-Tek, Potton, UK) at a wavelength of 490 nm. Cell survival rates were expressed as a percentage of the controls [52].

Iron-induced Stress on MIN6 Cells: Pancreatic MIN6 cells were pre-treated with various compounds before being exposed to rapid iron overload damage [17] with 50 μmol/L FAC and 20 μmol/L 8HQ (FAC + 8HQ). Cells were incubated for a further 2 h at 37 °C to allow rapid iron uptake. Then, cell viability was determined with the MTT assay.

Measurement of intracellular reactive oxygen species: Dihydrodichlorofluorescein (H2DCF) cell-permeant probe was used to monitor ROS according to the manufacturer’s recommendations. Pancreatic MIN6 cells from different treatment groups were collected and washed with fresh PBS and afterward incubated for 90 min in the dark at 37 °C in PBS with 10 μmol/L of H2DCF. The level of ROS generated was measured using flow cytometry based on the fluorescence intensity of DCF at 525 nm after excitation at 485 nm. The levels of ROS were shown as units of fluorescence compared with that of the control group.

Lipid peroxidation assay: The level of lipid peroxidation of MIN6 cells was measured and MDA content was determined using the lipid peroxidation colorimetric assay kit purchased from Cohesion Biosciences (London, UK) according to the manufacturer’s instructions. The absorbance of the supernatant was examined at 532 and 600 nm. MDA levels were normalized with protein content in cells and expressed as nmol/mg protein.

Intracellular iron measurement: MIN6 cells were plated in 6-well plates (10^6^ cells/well) and allowed to make an attachment for 4 to 5 days. Afterwards, MIN6 cells were incubated with different treatments. Cell pellets were collected and washed with fresh PBS, then re-suspended in 200 μL of 50 mM NaOH. Suspended cell pellets were dehydrated at 60 °C for 4 h. Subsequently, 400 μL of 69% HNO_3_ (nitric acid) for trace metal analysis was added to the samples. Agilent ICP MS 7700 series ICP MS instrument was used to measure iron levels under operating conditions suitable for routine multi-element analysis.

Glutathione assay: The cell GSH activity was evaluated by using a glutathione assay kit purchased from Sigma-Aldrich (Dorset, UK) according to the manufacturer’s instructions. The absorbance of the supernatants was read at 412 nm at a 1-min interval 5 times. The level of GSH was identified as nmol/mL.

SOD assay: SOD activity was detected by the superoxide dismutase assay kit (Sigma-Aldrich, Dorset, UK), according to the manufacturer’s instructions. This assay utilizes xanthine oxidase and hypoxanthine to generate superoxide radicals detected by tetrazolium salt. One unit of SOD is described as the amount of enzyme required to inhibit the dismutation of the superoxide radical by 50%. The results were shown as units per milliliter (U/mL) of SOD activity.

Insulin secretion assay: MIN6 β cells were seeded at a density of 4 × 10^6^ cells/well in a 12-well plate for 24 h at 37 °C in the incubator at 5% CO_2_. Cells were washed three times with glucose-free Krebs buffer, and then kept in 0.5% BSA Krebs buffer (2 mmol/L glucose) at 37 °C for 1 h in an atmosphere of 5% CO_2_. MIN6 insulinoma cells were again washed three times with glucose-free Krebs buffer. Afterwards, cells were maintained in 0.05% BSA Krebs buffer (20 mmol/L KCl or 2 mmol/L glucose) and treated with FA, FAS and FAC + 8HQ at certain doses in the presence of 20 mmol/L glucose for 3 h. For insulin measurements, supernatants with mouse or rat insulin were collected and the anti-rat/mouse insulin ELISA kit (Millipore; EZRMI-13K) was used as a standard in accordance with the manufacturer’s instructions. The absorbance was read at 450 nm using a microplate reader (Bio-Tek ELx800).

Real-time PCR analysis: Total RNA was extracted from tissues in all groups of mice using the Trizol reagent (Thermo Fisher Scientific, Swindon, UK), following the manufacturer’s protocols. RNA concentrations were measured spectrophotometrically using nanodrop, Hellma Tray-Cell Type 105.810 (Hellma Analytics, Southend on See, UK). Thermo Scientific Verso cDNA synthesis kit (Thermo Fisher Scientific, Swindon, UK) converted 1 μg of RNA from each sample into cDNA. Thermal cycling was run; initial denaturation; 95 °C for 5 min, followed by a set of 35 cycles of 95 °C for 30 s, Tm °C for 30 s and 72 °C for 45 s. Afterwards, the DNA extension time was allotted 5 min at 72 °C. The specificity of primers was verified by gel analysis and sequencing confirmation prior to their application for gene expression analysis; primers were used in Table 1. No product was observed in the minus-reverse transcriptase control sample; therefore, the samples were free from genomic DNA contamination.

Statistical Analysis: Statistical analysis was performed by GraphPad Prism 9.0 (GraphPad Software, San Diego, CA, USA) using one-way analysis of variance (ANOVA) and Tukey’s multiple comparisons post hoc test to compare the means of the experimental groups. The experiments were repeated in triplicate and each treatment had at least 8 replicates. All the values are presented as mean ± SEM. The test was considered significant when a *p* value was less than 0.05.

## 5. Conclusions

FA enhanced cell viability, decreased the accumulation of ROS and MDA accumulation and elevated glutathione levels in iron-loaded MIN6 cells. Moreover, the phenolics conferred protection against iron-loaded liver tissue of BALB/c mice via activating Nrf2, HO-1, NQO1, GCLC and GPX4 signalling pathway. FA protects pancreatic cells and the liver from iron-induced damage via the Nrf2 antioxidant activation mechanism, highlighting its potential therapeutic value for the treatment of T2D.

## Figures and Tables

**Table 1:** The RT-PCR specific primers.

Genes	Forward Primer	Reverse Primer
NFR2 (NM_010902)	catgatggacttggagttgc	cctccaaaggatgtcaatcaa
GPx4 (AB030643)	tttcctgacacagggttcact	cagcctggtctggtaagca
HO-1 (NM_010442)	agggtcaggtgtccagagaa	cttccagggccgtgtagata
NQO1 (BC004579)	agcgttcggtattacgatcc	agtacaatcagggctcttctcg
GCLC (BC019374)	agatgatagaacacgggaggag	tgatcctaaagcgattgttcttc
FPN (AF215637)	gggtttcttagaagcaggtatgc	ttctcagtgtacacacctattcaagtc
FtH (NM_010239.2)	gctgaatgcaatggagtgtg	cagggtgtgcttgtcaaaga
RPL19 (NM_009078)	ccacaagctctttcctttcg	ggatccaaccagaccttcttt

## Data Availability

The data presented in this study are available in this manuscript.

## Supplementary Materials

The following supporting information can be downloaded at: https://www.mdpi.com/article/10.3390/molecules28104084/s1, Figure S1: Iron accumulation in MIN6 cells treated with the different iron sources. Pancreatic MIN6 β cells were treated with 50 μmol/L FAC, 50 μmol/L FAC and 20 μmol/L 8HQ (FAC+8HQ), 50 μmol/L FeSO4, Fe-NTA and hemin for 2 h. Then, cellular iron concentrations were measured by inductively coupled plasma mass spectrometry (ICP-OES). # *p* < 0.05 control vs. treatment groups. One-way ANOVA, Tukey post-hoc test.

## Sample Availability

Samples of the compounds are available from the authors.

## Abbreviations

DMEM, Dulbecco’s Modified Eagle’s Medium; H2DCF, dihydrodichlorofluorescein; FA, ferulic acid; FAS, ferulic Acid 4-O-sulfate disodium salt; FAC, ferric ammonium citrate; FPN, ferroportin; FtH, iron storing protein ferritin H; 8HQ, 8-hydroxyquinoline; GCLC, glutamate-cysteine ligase catalytic subunit; GSH, glutathione peroxidase; GPX4, glutathione peroxidase 4; HO-1, heme oxygenase-1; ID, iron dextran; MDA, malondialdehyde; Nrf2, nuclear factor erythroid 2-related factor 2; MTT, 3-(4,5 dimethyl-2-thiazolyl)-2,5-diphenyltetrazolium bromide; NQO1, NAD(P)H quinone dehydrogenase 1; ROS, reactive oxygen species; SOD, superoxide dismutase; T2D, type 2 diabetes.

## References

[1] Eid R., Arab N.T.T., Greenwood M.T. (2017). Iron mediated toxicity and programmed cell death: A review and a re-examination of existing paradigms. Biochim. Biophys. Acta Mol. Cell Res..

[2] Rajpathak S.N., Crandall J.P., Wylie-Rosett J., Kabat G.C., Rohan T.E., Hu F.B. (2009). The role of iron in type 2 diabetes in humans. Biochim. Biophys. Acta Gen. Subj..

[3] Simcox J.A., McClain D.A. (2013). Iron and diabetes risk. Cell Metab..

[4] Cooksey R.C., Jouihan H.A., Ajioka R.S., Hazel M.W., Jones D.L., Kushner J.P., McClain D.A. (2004). Oxidative stress, β-cell apoptosis, and decreased insulin secretory capacity in mouse models of hemochromatosis. Endocrinology.

[5] Masuda Y., Ichii H., Vaziri N.D. (2014). At pharmacologically relevant concentrations intravenous iron preparations cause pancreatic beta cell death. Am. J. Transl. Res..

[6] Schieber M., Chandel N.S. (2014). ROS function in redox signaling and oxidative stress. Curr. Biol..

[7] Bo J., Xie S., Guo Y., Zhang C., Guan Y., Li C., Lu J., Meng Q.H. (2016). Methylglyoxal impairs insulin secretion of pancreatic β-cells through increased production of ROS and mitochondrial dysfunction mediated by upregulation of UCP2 and MAPKs. J. Diabetes Res..

[8] Kohgo Y., Ikuta K., Ohtake T., Torimoto Y., Kato J. (2008). Body iron metabolism and pathophysiology of iron overload. Int. J. Hematol..

[9] Bao W., Rong Y., Rong S., Liu L. (2012). Dietary iron intake, body iron stores, and the risk of type 2 diabetes: A systematic review and meta-analysis. BMC Med..

[10] Mendler M.H., Turlin B., Moirand R., Jouanolle A.M., Sapey T., Guyader D., le Gall J.Y., Brissot P., David V., Deugnier Y. (1999). Insulin resistance–associated hepatic iron overload. Gastroenterology.

[11] Green A., Basile R., Rumberger J.M. (2006). Transferrin and iron induce insulin resistance of glucose transport in adipocytes. Metabolism.

[12] Johansen J.S., Harris A.K., Rychly D.J., Ergul A. (2005). Oxidative stress and the use of antioxidants in diabetes: Linking basic science to clinical pratice. Cardiovasc. Diabetol..

[13] Montane J., Cadavez L., Novials A. (2014). Stress and the inflammatory process: A major cause of pancreatic cell death in type 2 diabetes. Diabetes Metab. Syndr. Obes..

[14] Valavanidis A.V.T. (2013). Plant polyphenols: Recent advances in epidemiological research and other studies on cancer prevention. Stud. Nat. Prod. Chem..

[15] Rahman I., Biswas S.K., Kirkham P.A. (2006). Regulation of inflammation and redox signaling by dietary polyphenols. Biochem. Pharmacol..

[16] Zduńska K., Dana A., Kolodziejczak A., Rotsztejn H. (2018). Antioxidant properties of ferulic acid and its possible application. Skin Pharmacol. Physiol..

[17] Messner D.J., Rhieu B.H., Kowdley K.V. (2013). Iron overload causes oxidative stress and impaired insulin signaling in AML-12 hepatocytes. Dig. Dis. Sci..

[18] Fang S., Yu X., Ding H., Han J., Feng J. (2018). Effects of intracellular iron overload on cell death and identification of potent cell death inhibitors. Biochem. Biophys. Res. Commun..

[19] Rainey N.E., Moustapha A., Saric A., Nicolas G., Sureau F., Petit P.X. (2019). Iron chelation by curcumin suppresses both curcumin-induced autophagy and cell death together with iron overload neoplastic transformation. Cell Death Discov..

[20] Leanderson P., Tagesson C. (1996). Iron bound to the lipophilic iron chelator, 8-hydroxyquinoline, causes DNA strand breakage in cultured lung cells. Carcinogenesis.

[21] Mancuso C., Santangelo R. (2014). Ferulic acid: Pharmacological and toxicological aspects. Food Chem. Toxicol..

[22] Kose T., Sharp P.A., Latunde-Dada G.O. (2022). Upregulation of Nrf2 Signalling and the Inhibition of Erastin-Induced Ferroptosis by Ferulic Acid in MIN6 Cells. Int. J. Mol. Sci..

[23] Gaschler M.M., Stockwell B.R. (2017). Lipid peroxidation in cell death. Biochem. Biophys. Res. Commun..

[24] Wang J., Wang H. (2017). Oxidative stress in pancreatic beta cell regeneration. Oxid. Med. Cell Longev..

[25] Tewari R.K., Bachmann G., Hadacek F. (2015). Iron in complex with the alleged phytosiderophore 8-hydroxyquinoline induces functional iron deficiency and non-autolytic programmed cell death in rapeseed plants. Environ. Exp. Bot..

[26] Vona R., Gambardella L., Cittadini C., Straface E., Pietraforte D. (2019). Biomarkers of oxidative stress in metabolic syndrome and associated diseases. Oxid. Med. Cell Longev..

[27] Puntarulo S. (2005). Iron, oxidative stress and human health. Mol. Asp. Med..

[28] Sakihama Y., Cohen M.F., Grace S.C., Yamasaki H. (2002). Plant phenolic antioxidant and prooxidant activities: Phenolics-induced oxidative damage mediated by metals in plants. Toxicology.

[29] Crozier A., Jaganath I.B., Clifford M.N. (2009). Dietary phenolics: Chemistry, bioavailability and effects on health. Nat. Prod. Rep..

[30] Srinivasan M., Sudheer A.R., Menon V.P. (2007). MVP. Ferulic acid: Therapeutic potential through its antioxidant property. J. Clin. Biochem. Nutr..

[31] Bacanli M., Aydin S., Taner G., Göktaş H.G., Şahin T., Başaran A.A., Başaran N. (2014). The protective role of ferulic acid on sepsis-induced oxidative damage in Wistar albino rats. Environ. Toxicol. Pharmacol..

[32] Bellezza I., Giambanco I., Minelli A., Donato R. (2018). Nrf2-Keap1 signaling in oxidative and reductive stress. Biochim. Biophys. Acta Mol. Cell Res..

[33] Evans J.L., Goldfine I.D., Maddux B.A., Grodsky G.M. (2003). Are oxidative stress− activated signaling pathways mediators of insulin resistance and β-cell dysfunction?. Diabetes.

[34] Zhao Z., Moghadasian M.H. (2008). Chemistry, natural sources, dietary intake and pharmacokinetic properties of ferulic acid: A review. Food Chem..

[35] Nomura E., Kashiwada A., Hosoda A., Nakamura K., Morishita H., Tsuno T., Taniguchi H. (2003). Synthesis of amide compounds of ferulic acid, and their stimulatory effects on insulin secretion in vitro. Bioorg. Med. Chem..

[36] Fumeron F., Péan F., Driss F., Balkau B., Tichet J., Marre M., Grandchamp B., DESIR Study Group (2006). Ferritin and transferrin are both predictive of the onset of hyperglycemia in men and women over 3 years: The data from an epidemiological study on the Insulin Resistance Syndrome (DESIR) study. Diabetes Care.

[37] Ferńandez-Real J.M., Mcclain D., Manco M. (2015). Mechanisms Linking Glucose Homeostasis and Iron Metabolism Toward the Onset and Progression of Type 2 Diabetes. Diabetes Care.

[38] Krisai P., Leib S., Aeschbacher S., Kofler T., Assadian M., Maseli A., Todd J., Estis J., Risch M., Risch L. (2016). Relationships of iron metabolism with insulin resistance and glucose levels in young and healthy adults. Eur. J. Intern. Med..

[39] Hansen J.B., Moen I.W., Mandrup-Poulsen T. (2014). Iron: The hard player in diabetes pathophysiology. Acta Physiol..

[40] Abraham D., Rogers J., Gault P., Kushner J.P., McClain D.A. (2006). Increased insulin secretory capacity but decreased insulin sensitivity after correction of iron overload by phlebotomy in hereditary haemochromatosis. Diabetologia.

[41] Basu T., Panja S., Shendge A.K., Das A., Mandal N. (2018). A natural antioxidant, tannic acid mitigates iron-overload induced hepatotoxicity in Swiss albino mice through ROS regulation. Environ. Toxicol..

[42] Liao X., Zheng S., Lu K., Xiao X., Wu S., Ming J. (2016). Plant Polyphenols Exert Antioxidant Activity of by Nrf2/ARE Signaling Pathway–A Review. Food Sci..

[43] Kaspar J.W., Niture S.K., Jaiswal A.K. (2009). Nrf2:INrf2 (Keap1) signaling in oxidative stress. Free Radic. Biol. Med..

[44] Tang X., Liu J., Yao S., Zheng J., Gong X., Xiao B. (2022). Ferulic acid alleviates alveolar epithelial barrier dysfunction in sepsis-induced acute lung injury by activating the Nrf2/HO-1 pathway and inhibiting ferroptosis. Pharm. Biol..

[45] Qi G., Mi Y., Fan R., Li R., Wang Y., Li X., Huang S., Liu X. (2017). Tea polyphenols ameliorate hydrogen peroxide-and constant darkness-triggered oxidative stress via modulating the Keap1/Nrf2 transcriptional signaling pathway in HepG2 cells and mice liver. RSC Adv..

[46] Tian C., Zhao J., Xiong Q., Yu H., Du H. (2023). Secondary iron overload induces chronic pancreatitis and ferroptosis of acinar cells in mice. Int. J. Mol. Med..

[47] Reagan-Shaw S., Nihal M., Ahmad N. (2008). Dose translation from animal to human studies revisited. FASEB J..

[48] Bourne L.C., Rice-Evans C. (1998). Bioavailability of ferulic acid. Biochem. Biophys. Res. Commun..

[49] Anson N.M., van den Berg R., Havenaar R., Bast A., Haenen G.R. (2009). Bioavailability of ferulic acid is determined by its bioaccessibility. Bioavailability of ferulic acid is determined by its bioaccessibility. J. Cereal Sci..

[50] Kose T., Vera-Aviles M., Sharp P.A., Latunde-Dada G.O. (2019). Curcumin and (−)-epigallocatechin-3-gallate protect murine MIN6 pancreatic beta-cells against iron toxicity and erastin-induced ferroptosis. Pharmaceuticals.

[51] Miyazaki J.I., Araki K., Yamato E., Ikegami H., Asano T., Shibasaki Y., Oka Y., Yamamura K.I. (1990). Establishment of a pancreatic β cell line that retains glucose-inducible insulin secretion: Special reference to expression of glucose transporter isoforms. Endocr. J..

[52] Zhang S., Ntasis E., Kabtni S., van den Born J., Navis G., Bakker S.J., Krämer B.K., Yard B.A., Hauske S.J. (2016). Hyperglycemia does not affect iron mediated toxicity of cultured endothelial and renal tubular epithelial cells: Influence of L-carnosine. J. Diabetes Res..

