# Supplementary material for: Phenolic Acids Rescue Iron-Induced Damage in Murine Pancreatic Cells and Tissues
Source: Molecules. 2023 May 14;28(10):4084. doi: 10.3390/molecules28104084 (PMC10224177; doi:10.3390/molecules28104084)
Supplement: Supplementary file 1 [file molecules-28-04084-s001.zip › molecules-2347120-supplementary.pdf]

Supplementary Figure

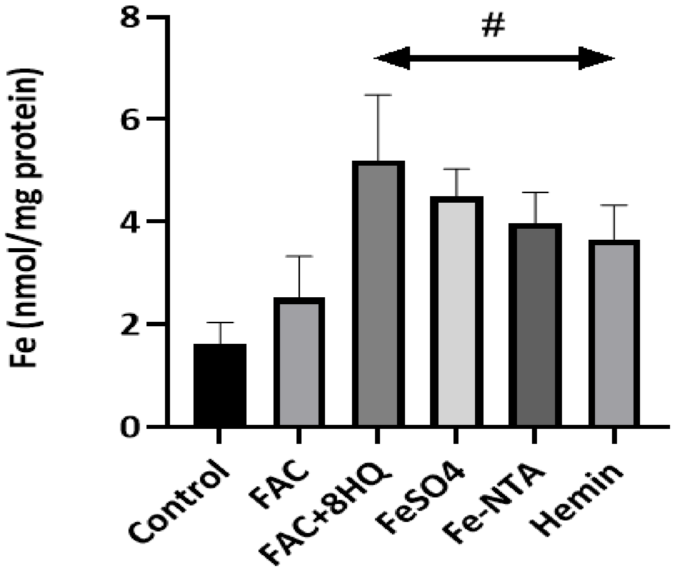

Figure S1: Iron accumulation in MIN6 cells treated with the different iron sources. Pancreatic MIN6 β cells were treated with 50 µmol/L FAC, 50 µmol/L FAC and 20 µmol/L 8HQ (FAC+8HQ), 50 µmol/L FeSO4, Fe-NTA and hemin for 2 h. Then, cellular iron concentrations were measured by inductively coupled plasma mass spectrometry (ICP-OES). # $p < 0.05$ control vs. treatment groups. One-way ANOVA, Tukey post-hoc test.
